# 595 Hypochlorous Acid for Skin Preparation in Fractional Ablative CO2 Laser Treatment of Burn Scars

**DOI:** 10.1093/jbcr/irac012.223

**Published:** 2022-03-23

**Authors:** Jenna C Bekeny, Jeffrey W Shupp, Taryn E Travis

**Affiliations:** MedStar Georgetown University Hospital, Washington, District of Columbia; MedStar Washington Hospital Center, Washington DC, District of Columbia; MedStar Washington Hospital Center, Washington, District of Columbia

## Abstract

**Introduction:**

While complications from fractional ablative CO2 laser treatment for burn hypertrophic scar (HTS) are relatively rare, the risk of surgical site infection (SSI) after laser treatment exists. When infection occurs, it has been linked to further morbidity, such as scar development in previously unaffected skin. Hypochlorous acid is a non-flammable and colorless antiseptic solution that has been used to treat wounds and is believed to be a less cytotoxic alternative to other agents such as betadine and chlorhexidine. This study aims to investigate the use of hypochlorous acid solution as a skin prep for patients undergoing laser scar revision for HTS and determine its efficacy in preventing SSI.

**Methods:**

All patients undergoing fractional ablative CO2 laser revision of HTS at a single center from September 2016 through September 2021 were retrospectively reviewed. Data pertaining to patient demographics, skin characteristics, treatment sessions, and post-treatment complications were recorded. All patients underwent topical skin preparation with hypochlorous acid-soaked laparotomy pads for 10 minutes before laser treatment. No perioperative oral or topical antibiotics were given to any included patient. Patients were excluded from analysis if they were not treated with a fractional ablative CO2 laser, were treated for a scar etiology outside of burn injury, or if insufficient follow-up was documented.

**Results:**

A total of 208 patients undergoing a total of 758 laser treatments were identified. Average patient age was 45 years (SD 16), with 112 (53.8%) patients being female and 96 (46.2%) being male. Median number of laser treatments was 3 (IQR: 1-5). Patients from all Fitzpatrick skin types were treated, with the most common type being type 5 (n=54, 26.0%). None (n=0, 0.0%) of the 208 patients experienced a surgical site infection after any of the laser treatments received.

**Conclusions:**

Hypochlorous acid is a safe and effective skin preparation for patients undergoing laser burn-scar revision to prevent SSI. Moreover it is non-flammable, colorless and does not need to dry prior to procedure.

